# Carbon and fullerene nanomaterials in plant system

**DOI:** 10.1186/1477-3155-12-16

**Published:** 2014-04-25

**Authors:** Azamal Husen, Khwaja Salahuddin Siddiqi

**Affiliations:** 1 Department of Biology, College of Natural and Computational Sciences, University of Gondar, P.O. Box 196, Gondar, Ethiopia; 2 Department of Chemistry, College of Natural and Computational Sciences, University of Gondar, P.O. Box 196, Gondar, Ethiopia

**Keywords:** Carbon nanomaterials, Uptake, Accumulation, Translocation, Plant growth, Seed germination, Agriculture

## Abstract

Both the functionalized and non functionalized carbon nanomaterials influence fruit and crop production in edible plants and vegetables. The fullerene, C_60_ and carbon nanotubes have been shown to increase the water retaining capacity, biomass and fruit yield in plants up to ~118% which is a remarkable achievement of nanotechnology in recent years. The fullerene treated bitter melon seeds also increase the phytomedicine contents such as cucurbitacin-B (74%), lycopene (82%), charantin (20%) and insulin (91%). Since as little as 50 μg mL^−1^ of carbon nanotubes increase the tomato production by about 200%, they may be exploited to enhance the agriculture production in future. It has been observed that, in certain cases, non functionalized multi-wall carbon nanotubes are toxic to both plants and animals but the toxicity can be drastically reduced if they are functionalized.

## Introduction

History of carbon is difficult to trace although it is as old as human civilization. Charcoal was used for water purification and adsorbent by ancient Hindus in India, and wood charcoal as an adsorbent and purifying agent by Egyptians and Sumerians as early as 3750 to 1500BC [1]. ‘Indian inks’ made from soot were used in the oldest Egyptian herioglyps on papyrus. Although activated charcoal, an allotrope of carbon is generally used as decolorizing agent in metallurgical operation, the use of carbon nanomaterials has been recognised in the recent years. A wide variety of carbon-based nanomaterials such as fullerene, fullerene cages, single-wall carbon nanotubes (SWCNTs) and multi-wall carbon nanotubes (MWCNTs) have been prepared. Diameters of SWCNTs and MWCNTs are typically between 0.8 to 2 nm and, 5 to 20 nm respectively, although MWCNTs diameters can exceed 100 nm. CNTs length ranges from less than 100 nm to several cm, thereby bridging molecular and macroscopic scales. Fullerenes are cage-like structures comprising of twelve 5-member carbon and unspecified 6-member rings in defect-free form. Even though an icosahedrally symmetrical structure (nC60) is the most commonly encountered fullerene, both smaller fullerene such as C28 and C36 and very large spherical fullerene conformations have been identified and characterized [2,3].

Carbon nanomaterial and fullerenes have been used as conducting material, optical devices, quantum computer, removal of biological contaminants, molecular switch, tissue engineering, pharmacy and medicine or carrier in drug delivery system [4-11]. Fullerenes and their derivatives are known to be powerful antioxidant in vivo (without much toxicity) and neuroprotecting agents [12-14]. The antioxidant, antiviral and anti cancerous activity of fullerols, C_60_(OH)_20_ has been ascribed to suppressed accumulation of superoxide and hydroxyl radical-initiated lipid peroxidation and free radical scavenging.

Carbon nanotubes - field effect transistors have been shown for selective detection of oxidase, dehydrogenase activity and many other enzymes and biomolecules, although it has some limitation [15,16]. Carbon nanotubes have been proposed as scaffolding agent for antimicrobial silver nanoparticles [17]. The optical properties of semiconducting SWCNTs has also been explored in photoluminescent detection of protein and selective biomolecules [18-23]. The unfunctionalized raw MWCNTs have been demonstrated to be carcinogenic to mice [24] while appropriately functionalized ones, did not show any apparent toxicity both in vitro and in vivo [25-28]. There are many factors which appear to be responsible for the toxicity of carbon nanotubes for instance, (a) hydrophobic nature of nanotubes (b) presence of catalyst and (c) presence of surfactants. Functionalized carbon nanotubes have been used in drug delivery. The cavity provided by hollow nanotubes allows large molecules like mellollocence, complex ions, fullerenes and DNA to be encapsulated [29-31] and delivered to the target cells. It is paradoxical and requires long time experiment before practically putting it to use in living system as a carrier. Application of carbon nanomaterials in agriculture is the basic need of the hour because of increasing population and depleting resources. In the recent years, carbon nanomaterials are used in agriculture to increase the crop production (Table 1). While they are known to be useful in (many cases) seed germination, root growth and photosynthesis there are many other aspects like; uptake and rejection, accumulation and transportation and transmission of nanomaterial in the progeny. If any genetic damage occurs due to forced injection of carbon nano material, it may cause mutation which may be transmitted to the next generation of the plant and, may have unwarranted influence in them. Although, a rapid progress is made in the synthesis and use of carbon nanomaterial their mechanism of interaction with plant is not well understood because conflicting reports from various quarters have been received [32-35].

**Table 1:** Effects of carbon nanomaterials on plants

**Nanoparticle**	**Size (nm)**	**Plant**	**Concentration**	**Effect**	**References**
C_60_ Fullerenes	1450-1900	Corn	500 mg kg^−1^	Reduced biomass	Torre-Roche et al. [36]
		Soybean		Reduced biomass	Torre-Roche et al. [36]
Fullerol [C_60_ (OH)_20_]	1.5 ± 0.2-5.00 ± 0.7	Bitter melon	0.943, 4.72, 9.43, 10.88 and 47.20 nM	Increased biomass yield, water content, fruit length, fruit number, and fruit fresh weight, increased two anticancerous phytomedicines, cucurbitacin-B and lycopene, and two antidiabetic phytomedicines, charantin and insulin	Kolle et al. [37]
Functionalized carbon nanotube	8	Lettuce	104, 315, 1750 mg L^−1^	Reduced root length at longer exposure	Cañas et al. [34]
Functionalized single-walled carbon nanotube	8	Cabbage, carrot, lettuce, onion, tomato	9, 56, 315, 1750 mg L^−1^	No effect	Cañas et al. [34]
Multiwalled carbon nanotube		Zucchini	1000 mg L^−1^	Reduced biomass	Stampoulis et al. [38]
		Lettuce	2000 mg L^−1^	Reduced root length	Lin and Xing [39]
	Diameter range: 10-30	Rice	20, 40, 80 mg L^−1^	Chromatin condensed inside the cytoplasm and caused cell death, plasma membrane detachment from cell wall and cell shrinkage	Tan and Lin [40]
		Tomato	10-40 mg L^−1^	Significant increase in germination rate, fresh biomass, and length of stem significantly enhanced moisture content inside tomato seeds	Khodakovskaya et al. [41]
		Corn, cucumber, radish, rapeseed, ryegrass, lettuce	2000 mg L^−1^	No effect on germination	Lin and Xing [39]
		Ryegrass	2000 mg L^−1^	Increased root length	Lin and Xing [39]
		Zucchini		No effect on the germination	Stampoulis et al. [38]
	Internal dimension: 110-170	Wheat	100 mg L^−1^	No significant effect on root or shoot growth	Wild and Jones [42]
	10-25	Tomato	50-200 μg L^−1^	Significant increase in plant height, flower and fruit formation	Khodakovskaya et al. [43]
Single-walled carbon nanotube	1.19 (major), 18, 722	Rice	400 mg L^−1^	Delayed flowering, decreased yield	Lin et al. [44]
	8	Tomato	104, 315, 1750 mg L^−1^	Most sensitive in root reduction	Cañas et al. [34]
	8	Cucumber onion,	104, 315, 1750 mg L^−1^	Increased root length	Cañas et al. [34]
	8	Cabbage, carrot, lettuce	104, 315, 1750 mg L^−1^	No effect	Cañas et al. [34]

Since carbon nanotubes can stimulate growth, gene and protein expression of aquaporin in tobacco cells [45] it may also trigger the reproductive genes in similar other plants. The penetration of CNTs into the plant system is inversely proportional to its size and, it is the key factor to increase the plant growth and fruits. Perhaps the large size of activated carbon particles are forbidden to enter the plant cell and therefore, get adsorbed on the surface.

In this review we are summarising the uptake and accumulation of carbon nanotubes, fullerenes and fullerol in edible and crop plants. Also, their effect on rate of germination, increase in biomass, absorption and translocation in different parts of plants has been assessed.

### Uptake, translocation and accumulation of carbon nanomaterials

All nanomaterials suspended in water may be selectively absorbed or rejected by plants but essential plant nutrients are generally absorbed. The carbon nanomaterials may however, be absorbed through roots of the plants but in seeds it may penetrate making a hole of appropriate size and translocated to the shoot. The absorption of carbon nanomaterial depends mainly on its interaction with suspended organic materials, its colloidal nature and the homo-heterogenous media which permit its smooth flow into the plant system. The natural organic matter coupled with MWCNTs and fullerene (C_70_) increases the hydrophilicity of nanomaterial. Carbon nanotubes are capable of penetrating plant seed and dramatically affect the seed germination and plant growth [45].

**Figure 1:**
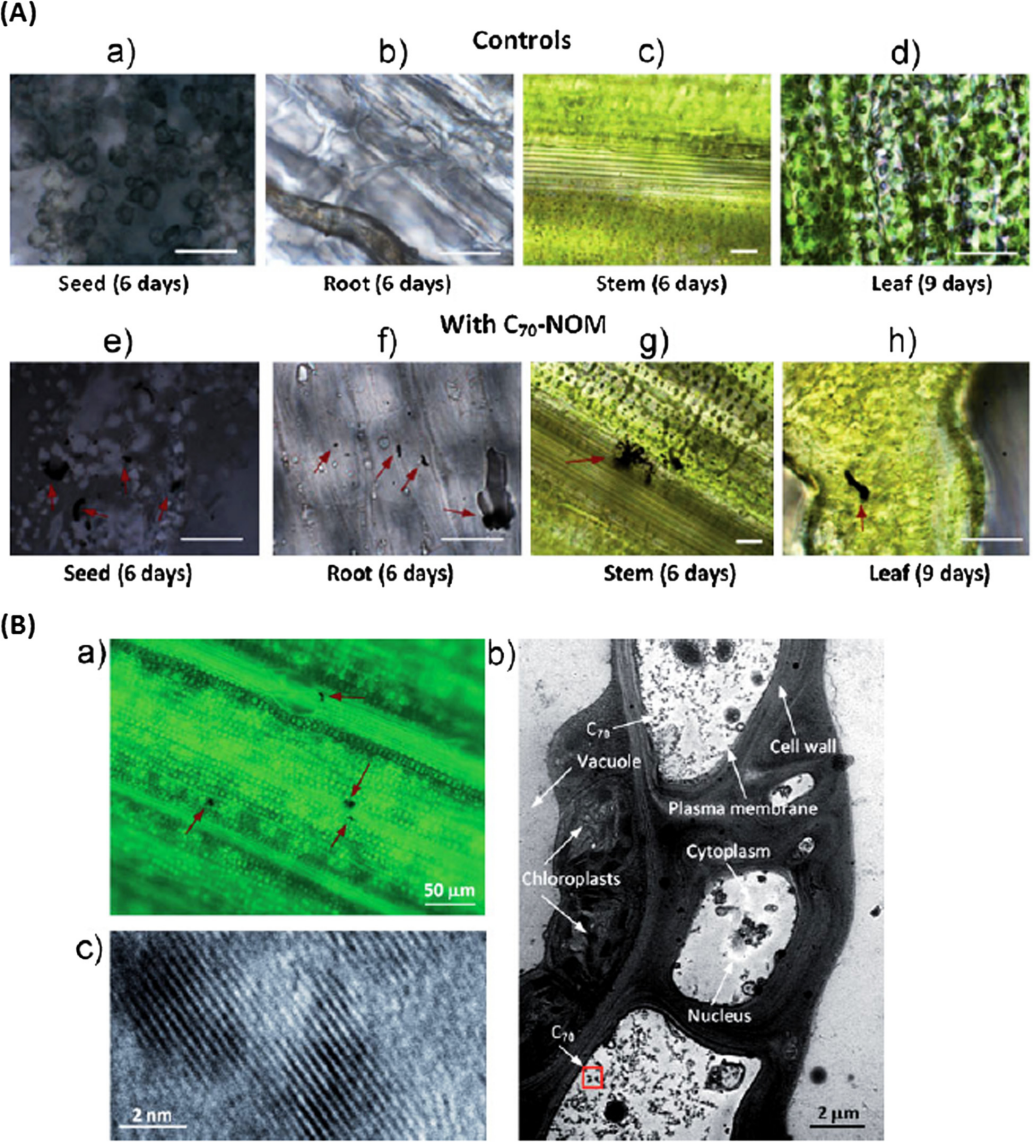
**Bright-field images of rice plants showing C**_**70 **_**uptake. (A)** Bright field images of root and leaf portions of 1-week-old rice seedlings. Control plants without any C_70_ (a–d) and treated plants showing C_70_ uptake (e and f). Arrows indicate the aggregation of nanoparticles in corresponding C_70_ treated plant tissues (scale bars are 20 μm). **(B)** (a) Bright field image of the leaf portion of a second generation rice plant. C_70_ aggregates were mostly found near the leaf vascular system. (b) TEM image of the leaf cells showing C_70_ particles (C_70_: 20 mg L^−1^). (c) TEM image of C_70_ particles with higher magnification [44].

It has been demonstrated that SWCNTs of length less than 500 nm labelled with fluorescein isothiocyanate (FITC) penetrate the cell wall of the living plants by endocytosis. FITC alone is not easily taken up by the plants [46,47] which means that both of them jointly facilitate the absorption/penetration of nanomaterials. Once the carbon nanomaterial is accumulated in the plant it may be transferred to the consumer. Tomato seedlings have been shown to absorb MWCNTs through seed and roots [41]. They enter the seed coat by piercing, increasing rapid absorption of water [42]. Carbon based nanomaterial can be taken up by the plants via root and distributed in the aerial parts. Their interaction with the chemicals present on the surface of root is not a driving force for their transport. They are transported by capillary action to places where the passage is wider than their size. When they reach a point where the passage is narrow carbon nanomaterial is accumulated and blocks the passage for nutrients to flow further. However, the carbon nanomaterial is genetically transmitted to the next generation [44,48]. The two consecutive generations of rice seeds grown showed the accumulation of C_70_ nanomaterial at different stages and different parts of the plant (Figure 1) followed by insertion of C_70_ nanomaterial in the first generation and then subsequent transmission to the progeny in the second generation. As a consequence of accumulation of nanomaterial of appropriate size in the plant cell the absorption/uptake of essential nutrients is hampered and hence the growth and flowering is delayed.

Serag et al. [11] have studied the effect of carbon nanomaterial at molecular level. They have shown that the diameter and length of SWCNTs are limiting factors for their penetration into the cell wall of the plants. If the size of carbon nanomaterial is too small it may diffuse and leak but if it is too large it may remain out of the cell and immobilised even if it has penetrated the plant cell wall. The chemically shortened SWCNTs have been shown to traverse through both cell wall and the cell membrane of tobacco and *Catharanthus*[46,49,50]. On the basis of high resolution transmission electron microscopy studies it has been shown that long MWCNTs (larger than 200 nm) get accumulated in subcellular organelles while the shorter ones (30-100 nm) were found into vacuoles, nucleus and plastids [11,51].

**Figure 2:**
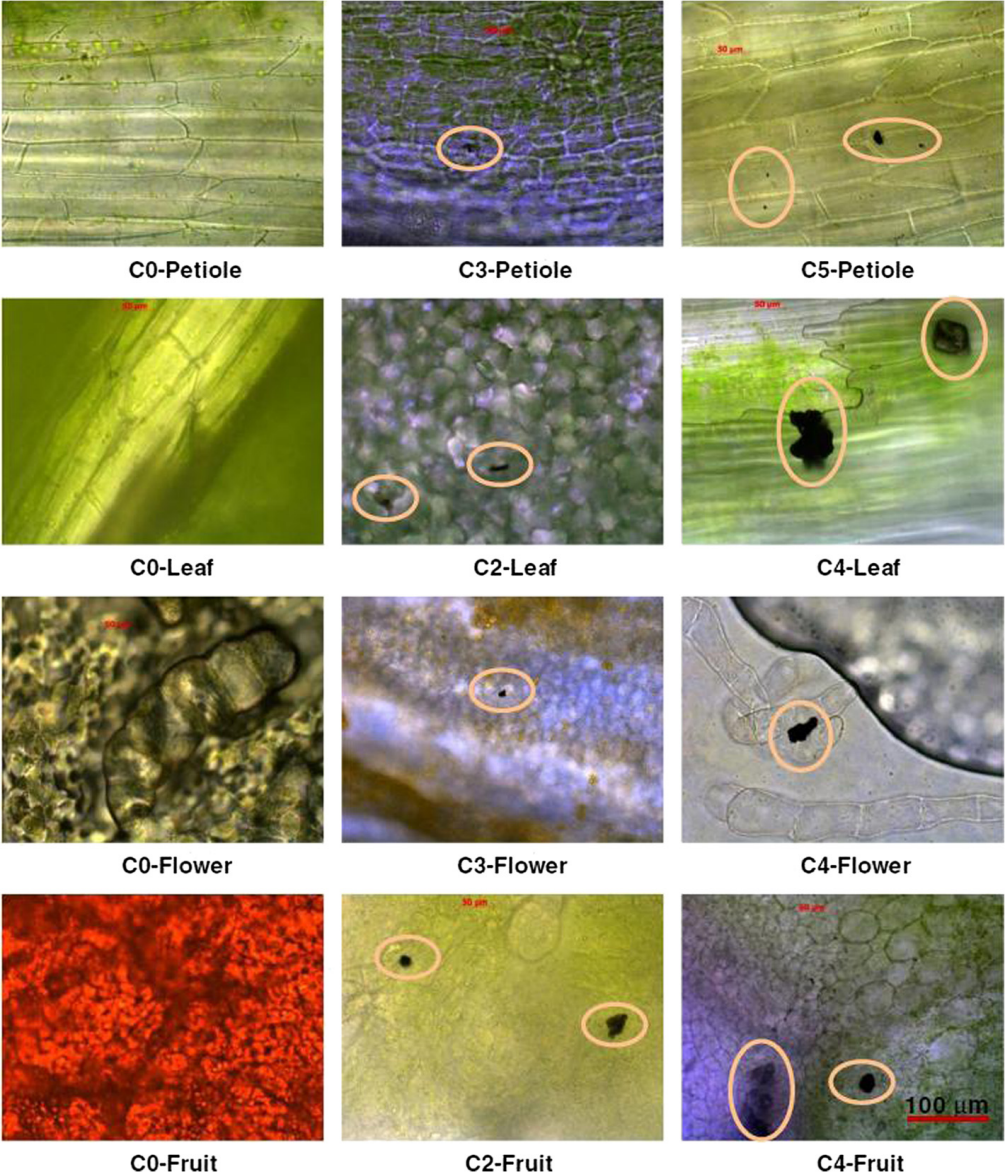
**Biodistribution of fullerols in plant organs including petioles, leaves, flowers, and fruits.** The circles highlight black aggregates which were later confirmed by FTIR as fullerols [37].

Kole et al. [37] have demonstrated in an experiment with bitter melon, for the uptake of carbon nanomaterial that, with increasing concentration the hydrodynamic size of fullerol increases due to extensive hydrogen bonding as shown below. Every part of the plant was found to contain fullerol (Figure 2) which was also confirmed by FTIR spectral data.

Raman spectroscopy was used to locate the presence of carbon nanotubes in the reproductive organ of tomato plant. Clusters of carbon nanotubes were detected in the flower by the appearance of a Raman peak at 1587 cm^−1^ which was missing in the plant treated with activated charcoal or the control. It has been already demonstrated [52] on the basis of Raman photochemical and photoacoustic studies, that carbon nanotubes reach the leaves and fruits of tomato plants, irrigated with different concentrations of carbon nanotubes. Such nanomaterial might act as growth regulators of plants and may also increase crop production in cereal plants. However, an assessment of the toxicity of carbon nanotubes on environment and human system needs investigation.

### Response of carbon nanomaterials on plant growth and yield

A large number of reports have shown the effect of carbon nanomaterials on both plant and mammalian cells [38,39,52-55]. It has been unanimously observed that highly functionalized carbon nanotubes exhibit remarkable reduction in toxicity [56,57] even in cultured plant cells [49,50,58,59].

#### Effect of fullerene

It was found that bitter melon seeds treated with fullerene increased the yield by 112 to 128%. However, with large concentration of C_60_ the effect diminishes. The highest biomass increased up to ~54%. It confirms the absorption and translocation of fullerol in plants. All the cucurbitacin-B (74%), lycopene (82%), charantin (20%) and insulin (91%) were found to increase with respect to control. The bitter melon seeds treated with five different concentration of fullerol showed an enhancement in biomass, fruit yield and phytomedicine contents [37]. Fullerol of an appropriate size and concentration may, therefore, be used to increase the yield of cereal and fruit crop, nevertheless the adverse effect on the environment must be assessed before recommending its application in general.

#### Effect of SWCNTs and MWCNTS

The effects of functionalized and non-functionalized SWCNTs on six plants namely, cabbage, carrot, cucumber, lettuce, onion and tomato were investigated by Cañas et al. [34]. Root elongation was observed in onion and cucumber with the formation of sheets of carbon nanotubes. It is surprising that neither of the carbon nanotubes entered the root of the plant. Root elongation in such plants is of no use because it is not edible. The cabbage and carrot were unaffected. Root elongation in lettuce and tomato was inhibited. Contradicting reports have been received regarding the influence of carbon nano tube on various plants. Khodakovskaya et al. [41] have shown, from their experiments on similar plants, that increased water uptake by seeds increased their germination.

Experiment on suspension rice cells with MWCNTs showed that it induced the production and accumulation of reactive oxygen species (ROS) as a consequence of which, plant cells die [60]. It also decreases the dry weight, chlorophyll content and the activity of superoxide dismutase (SOD) in *Arabidopsis* suspension cells [35]. The reason for all cell damage and eventual death is ascribed to apoptosis or necrosis. In case of interaction of MWCNTs with rice suspension, cells had aggregated in self defence to prevent the other cells from damage by them [60]. Such defensive mechanism sometime forces the plants to undergo certain changes in their activity such as thickening of the cell wall to avoid penetration by MWCNTs or other unwanted foreign particles. The rice seeds were germinated after their treatment with SWCNTs, MWCNTs and C_70_ in presence of nano-organic material and harvested after six months. They were further grown as second generation seed and examined for carbon nanomaterial in different parts of the plant. The carbon nanomaterials were found as black aggregate in the following chronological order:

Seed>root>stem>leaf

This proved that C_70_ is transmitted to the next generation of the plant which was further confirmed from IR and Raman spectral data. However, after sometime no C_70_ was present in root showing complete transport while uptake of MWCNTs was almost insignificant. It is normally accumulated in the root and delays the germination of rice seeds by 1 month [60].

It is known that MWCNTs do not have any significant effect on seed germination in a variety of plants even when treated for five days with as high concentration as 2000 mg L^−1^[39]. A similar observation was made by Wild and Jones [42] in case of wheat root. However, conflicting reports have been received about tomato seed germination with as little MWCNTs as 10–40 mgL^−1^[41]. Perhaps the effect of such nanomaterial on different plants is different but it may change in the presence of other substances or the organic material already present in the medium.

Khodakovskaya et al. [43] have demonstrated the influence of MWCNTs on the tomato plant from the germination to the flowering stage. They observed that when the tomato plants were treated separately with both activated charcoal and carbon nanotubes the height was increased in the plants treated with carbon nanotubes only. It is surprising to note that tomato plants treated with carbon nanotubes bore twofold flowers compared to the control and those treated with activated charcoal. It shows potential of increasing the tomato production by 200%. Since at 50 μg mL^−1^ dose it had the same influence as that obtained by 200 μg mL^−1^, a minimum dose of 50 μg mL^−1^ may be fixed. It may be inferred from the findings that carbon nanotubes influence the reproductive system although the mechanism is yet to be understood.

The maize seedlings grown in agar gel, treated by different concentrations of pristine MWCNTs, had dramatic influence on water uptake and growth [61]. Analysis of seedlings showed the presence of nearly all elements of 3^rd^ (Na, Mg, Al, Si, P, S and Cl) and 4^th^ (K, Ca, Ga, Ge, As, Se and Br) period without much variation when compared with control or minimum MWCNTs concentration of 5 mgL^−1^. The authors have shown changes in the morphology of MWCNTs after the addition of Fe^2+^ and Fe^3+^ in separate experiments. Since Fe^2+^ in aqueous medium is immediately oxidised to Fe^3+^, the effect of addition of Fe^2+^ will exactly be the same as that of Fe^3+^. However, Fe^2+^ can be stabilized in acidic medium.

In one of the experiments Mondal et al. [62] have shown that MWCNTs of approximately 30 nm diameter enhance the rate of germination and growth of *Brassica juncea*. Likewise, TiO_2_ has also been reported to enhance the rate of germination and strength of spinach seedlings [63]. Later it was found that [41] such nanoparticles increase the moisture content of the seeds, perhaps owing to adsorption, which in turn, may be absorbed by the seedling. The oxidized carbon nanotubes had better effect on the seed germination than the carbon nanotubes alone, although the concentration of the oxidized carbon nanotubes was much lower. Quite good results were obtained with oxidized MWCNTs (2.3x10^−3^ mg mL^−1^) but when the concentration exceeds 46x10^−3^ mg mL^−1^ both pure and oxidized MWCNTs inhibit the germination of mustard seeds. It clearly indicated that the rate of growth is concentration dependent. This technique may, therefore, be applied to increase the rate of germination of crop plants.

It has been suggested that electrical conductivity of a solution increases when plant tissues are immersed in carbon nanomaterials. This is correct up to a certain limit above which the conductance becomes constant because, as the concentration [63] of leached salts, amino acids, potassium, phosphate, sugar and carbohydrates increases, the freedom of movement of these molecules and ions decreases. Aquaporins are water channels which selectively allow water molecules to flow in and out of the tissue but also reject certain substances in order to maintain equilibrium. It is concluded that pre -soaking of seeds with very low concentration of oxidized MWCNTs have positive effect on seed germination.

#### Effect of industrial grade MWCNTs

Taunith, a carbon nanotubes material containing industrialized MWCNTs has been shown to stimulate the growth of *Onobrychis arenaria* plant and enhance peroxidase activity [64]. It suggests that the accumulation and onward translocation of CNTs in leaves and roots occurs. The increase in peroxidase activity is associated with the oxidative stress caused by CNTs. Elena et al. [64] have shown that MWCNTs accumulated at the root surface quite often penetrate the epidermal cells causing injury as a result of which the level of peroxidase activity is elevated. The accumulation of CNTs has been confirmed from TEM images of different parts of the plant.

Recently, Miralles et al. [65] have studied the effect of industrial grade MWCNTs (2560 mg L^−1^) and their impurities on alfalfa and wheat germination. The nanomaterial was functionalized with Fe_3_O_4_ nanoparticles in plant tissue. It was observed that carbon nanotube is adsorbed on to the root surface with significant root elongation in both alfalfa and wheat but with insignificant uptake and translocation [65].

It is important to note that in some cases [66,67] the carbon nanotubes were mixed with catalysts such as Al_2_O_3_ and Fe, suspected to contain Feº, Fe_2_O_3_, Fe_3_O_4_ or dissolved Fe^2+^ or Fe^3+^ ions. It should be remembered that iron metal as nanoparticle may be present but Fe^2+^/Fe^3+^ may be there only in equilibrium with their anions like Cl^−^, Br^−^, I^−^, SO_4_^2−^, CO_3_^2−^, CH_3_COO^−^ and similarly the oxides of iron such as FeO, Fe_2_O_3_ and Fe_3_O_4_ are equally likely to be present. On the other hand Al_2_O_3_ in water can persist only as Al(OH)_3_ according to the following reaction:

$${\mathrm{Al}}_{2}O_{3}{+}_{3}H_{2}O\to 2\mathrm{Al}{\left(\mathrm{OH}\right)}_{3}$$

The authors [65] suggest that their carbon nanotubes contain Fe/Al_2_O_3_ and the FTIR exhibits a peak at 1560 cm^−1^ showing υ(C = C) for graphite structure. The carbon or carbon nanotubes, in elemental form can not exhibit υ(C = C) because IR spectrum can detect the frequency of functional groups not that of an element. This υ(C = C) at 1560 cm^−1^ indicates the presence of some organic compound which remained undetected. If this peak is assigned to υ(C = C), it confirms the presence of alkene such as acetylene or ethylene or their derivative. The ester group indicated is actually a ketonic group (R_2_C = O). The ester group is –COOR and hence, the assignment is ambiguous. It has been shown in this case [65] that the rate of increased germination is mainly due to contamination and not due to carbon nanotubes alone. The enhanced wheat germination rate with respect to that of alfalfa has been ascribed to ease of penetration of carbon nanotube in the seed along with water. Since wheat seed is relatively softer than that of alfalfa the penetration and bioavailability of carbon nanotube in alfalfa is slow and hence the effect is low and delayed. Cañas et al. [34] have shown that carbon nanotube enhances the root growth of cucumber and onion seeds but affects adversely in tomato, lettuce, carrot and cabbage. It has also been suggested that carbon nanotube in tomato altered the gene expression thereby activating the growth [34]. The impurity (Fe or Fe_3_O_4_) in carbon nanotube influences the plant growth. Although Fe nanoparticles are not toxic to pumpkin [68,69] they inhibit the germination and growth in flax, ryegrass and barley [70].

Pristine carbon nanotubes have been found to induce toxicity in plants. SWCNTs are known to produce toxicity in rice and *Arabidopsis* leading to the death of ~ 25% of protoplast in 6 h [55]. MWCNTs were found to reduce the biomass of *Cucurbita pepo* plants [38]. However, effort is to be made to prevent the damage of plant cell from carbon nanomaterials [38]. Effect of carbon nanotubes on growth and development of tomato seedling germinated in Murashige and Skoog medium has been thoroughly studied [41] using TEM, TGA and Raman spectroscopic data in a concentration range between 10–40 μg mL^−1^.

#### Carbon nanotubes and pesticides

Effect of carbon nano materials on pesticide residue in zucchini, corn, tomato and soybean has been investigated by Torre-Roche et al. [36]. It was found that pesticide residue uptake by the above plants was reduced in presence of carbon nanotubes. Different parts of the plant had different quantum of chlordane and DDT accumulated which were reduced when exposed to MWCNTs. The quantity of it varies with species to species, dispersion, mobility and transport of the nanomaterials. Selective carbon nano materials in controlled quantity are capable of activating physiological processes in plants. Root growth in onion, cucumber and ryegrass and plant growth of tomato [34,39,52,71] has been shown by carbon nanotubes. They are also helpful in slow release of pesticides [72].

## Conclusion

Carbon/fullerene nanotechnology is a rapidly growing area of research which finds use in plant, medicine and engineering. Carbon nanotubes (single-wall carbon nanotubes and multi-wall carbon nanotubes) in many cases can penetrate the seed coat and plant cell wall which depends on their size, concentration and solubility. The size of carbon nanotubes alone is of great significance in agriculture and biotechnology. The penetration of carbon nanotubes into the plant system can bring changes in metabolic functions leading to an increase in biomass, fruit/grain yield. The nanobiotechnology may be helpful for the advancement of agriculture and plant sciences. Although in some cases, carbon nanomaterials are known to be phytotoxic their concentration may be controlled within the permissible limit to prevent any damage. Their bioavailability must be assessed if they pose risk to man and animals. The future prospect of carbon nanomaterials is fairly bright as it is a low cost solution to increase the crop production and fruit manifold.

## Competing interests

The authors declare that they have no competing interests.

## Authors’ contributions

AH gathered the research data. AH and KSS analyzed these data findings and wrote this review paper. Both authors read and approved the final manuscript.
